# Dispersion relation data for methylammonium lead triiodide perovskite deposited on a (100) silicon wafer using a two-step vapour-phase reaction process

**DOI:** 10.1016/j.dib.2015.10.026

**Published:** 2015-11-06

**Authors:** Laurie J. Phillips, Atef M. Rashed, Robert E. Treharne, James Kay, Peter Yates, Ivona Z. Mitrovic, Ayendra Weerakkody, Steve Hall, Ken Durose

**Affiliations:** a Stephenson Institute for Renewable Energy, University of Liverpool, Liverpool L69 7ZF, UK; b Department of Electrical Engineering and Electronics, University of Liverpool, Brownlow Hill, Liverpool L69 3GJ, UK

## Abstract

Ellipsometry was used to measure the amplitude ratio and phase difference of light undergoing a phase shift as it interacts with a thin film of organic–inorganic hybrid perovskite CH_3_NH_3_PbI_3_ (MAPI) deposited onto a (100) silicon wafer. The refractive index and extinction coefficient was extracted from a multi-oscillator model fit to the ellipsometry data, as a function of wavelength, from 300 to 1500 nm.

**Specifications table**

**Table:** 

Subject area	*Physics*
More specific subject area	*Lead*–*halide hybrid perovskite solar cells*
Type of data	*Comma separated variable file*
How data was acquired	*Ellipsometer*
Data format	*.csv file with three columns – wavelength (nm); n; k Unprocessed, and without interpolation, after taking the values from the fit.*
Experimental factors	*Samples were used as deposited without further treatment*
Experimental features	*A MAPI film was deposited onto a silicon wafer using a two-step vapour reaction phase process and a variable-angle ellipsometer used to take amplitude and phase change measurements.*
Data source location	*Liverpool, UK*
Data accessibility	*With article*

**Value of the data**

• The dispersion relations in this brief can be used in optical models to predict the transmission of light through a perovskite layer, particularly of use in combination with similar data from partner layers.
• Features in the data curves indicate transitions between energy levels. Therefore, this data can be compared to similar data taken from material fabricated using different methods and substrates.
• As the MAPI layer was deposited onto silicon, this data should prove particularly useful in investigating multi-junction tandem devices, combining hybrid lead–halide perovskites with silicon.

## 1 Data

The values presented here are the dimensionless refractive index and extinction coefficient of MAPI as a function of wavelength.

## 2 Experimental design, materials and methods

A 0.8 M lead iodide solution in DMF was heated to 70 °C and spin-coated onto a clean (100) silicon wafer at 3000 rpm and dried at 70 °C. This was then placed into an evacuated tube, surrounded by methylammonium iodide (MAI) powder and heated to 150° for one hour. Annealing in the presence of MAI converted the yellow lead iodide layer into dark brown MAPI. The layer was measured as 350 nm thick with an rms roughness of 5 nm using an Ambios XP-200 profilometer [1].

Variable angle spectroscopic ellipsometry data from the MAPI layer was acquired using a J.A. Woollam 2000 M ellipsometer at 65°, 70°, and 75° angles from 300 to 1500 nm in 1 nm steps. A multi-oscillator model was fit to all three curves simultaneously, and minimised to achieve a mean squared error (MSE) of 10. From the oscillator model fit, the dispersion relations were extracted. All fitting was completed using CompleteEASE software, from J.A. Woollam.
